# Joint damage is amplified in rheumatoid arthritis patients with positive thyroid autoantibodies

**DOI:** 10.7717/peerj.4216

**Published:** 2018-01-04

**Authors:** Yu-Lan Chen, Jian-Zi Lin, Ying-Qian Mo, Jin-Jian Liang, Qian-Hua Li, Cheng-Jing Zhou, Xiu-Ning Wei, Jian-Da Ma, Ze-Hong Yang, Dong-Hui Zheng, Lie Dai

**Affiliations:** 1 Department of Rheumatology, Sun Yat-Sen Memorial Hospital, Sun Yat-Sen University, Guangzhou, China; 2 Zhongshan School of Medicine, Sun Yat-Sen University, Guangzhou, China; 3 Department of Radiology, Sun Yat-Sen Memorial Hospital, Sun Yat-Sen University, Guangzhou, China

**Keywords:** Rheumatoid arthritis, Thyroid autoantibodies, Radiographic joint damage

## Abstract

**Background:**

Autoimmune thyroid disease (AITD), which is characterized by an increased presence of thyroid autoantibodies (TAbs), such as antibodies against thyroid peroxidase (TPOAbs) and antibodies against thyroglobulin (TgAbs), has been reported to be associated with rheumatoid arthritis (RA) because AITD and RA both involve autoimmunity. However, few data are available on the incidence of TAbs in Chinese RA patients, and studies on the association between TAbs and joint damage as well as synovitis in RA patients remain sparse. Here, we aimed to evaluate the incidence of TAbs in a consecutive Chinese RA cohort and to investigate whether the elevated presence of TAbs is associated with joint damage and synovitis in RA patients.

**Methods:**

A total of 125 hospitalized RA patients were consecutively recruited. Clinical data and available synovial tissues were collected at baseline, and TAbs and thyroid function were detected by chemiluminescent immunoassay. Patients who tested positive for TPOAbs or TgAbs were classified as the TAbs-positive group, and patients who tested positive for neither TPOAbs nor TgAbs were recruited as the TAbs-negative group. Disease activity was assessed using DAS28-ESR (the disease activity score in 28 joints and including the erythrocyte sedimentation rate). X-ray assessment of the hand/wrist was performed according to the Sharp/van der Heijde-modified Sharp score (mTSS), and patients with an mTSS score >10 were defined as having radiographic joint damage (RJD). Serial tissue sections were stained immunohistochemically for CD3, CD15, CD20, CD34, CD38, and CD68, and synovitis were assessed according to Krenn’s synovitis score.

**Results:**

A total of 44 (35%) patients were positive for either TPOAbs or TgAbs. Importantly, there was a significantly greater percentage of patients with RJD in the TAbs-positive group versus the TAbs-negative group (68% vs. 42%, *p* = 0.005). Compared with the TAbs-negative group, significantly more CD38-positive plasma cells infiltrated the TAbs-positive synovium, and a higher percentage of patients with high-grade synovitis were observed in the TAbs-positive group (5/8, 63% vs. 5/14, 36%). Moreover, RF positivity and disease activity indicators, including TJC28, DAS28-ESR, and CDAI, were significantly higher in the TAbs-positive group (all *p* < 0.05). Adjusted logistic regression analysis revealed that positive TAbs (OR 2.999, 95% CI [1.301–6.913]; *p* = 0.010) and disease duration (OR 1.013, 95% CI [1.006–1.019]; *p* < 0.001) were independently associated with RJD, and an odds ratio of 2.845 (95% CI [1.062–7.622]) was found for RJD in women with positive TAbs (*n* = 37) compared with those without TAbs (*n* = 59) (*p* = 0.038).

**Conclusion:**

Our data showed that joint destruction was amplified in RA patients with an elevated presence of TAbs, which supports the importance and necessity of TAbs and thyroid function screening and monitoring in RA patient management in clinical practice.

## Introduction

Rheumatoid arthritis (RA) is a systemic autoimmune disease that is characterized by joint destruction and deformity, affecting millions of people worldwide (McInnes & Schett, 2011). However, RA is more than a symmetrical inflammation of the joints, as increasing evidence supports a higher risk of other autoimmune disorders. Autoimmune thyroid disease (AITD) is one of the most common organ-specific autoimmune diseases, which is characterized by endocrine abnormalities and an elevated presence of thyroid antibodies (TAbs), such as antibodies against thyroid peroxidase (TPOAbs) and antibodies against thyroglobulin (TgAbs). Indeed, the coexistence of RA and AITD has been a subject of interest for several decades (Hijmans et al., 1961; Becker, Ferguson & McConahey, 1963). The two diseases appear to be associated based on their similarities in both genetics, such as HLA-DR B1, CTLA4 and PTPN22 (Raychaudhuri, 2010; Tomer & Huber, 2009), and environmental risk factors, such as infection, vaccines and smoking (De Carvalho, Pereira & Shoenfeld, 2009).

The prevalence of AITD in RA patients investigated in different studies varies considerably among countries, ranging from 0.5% in Morocco (Benamour et al., 1992) to 27% in Slovakia (Lazurova et al., 2009). A higher frequency of AITD or TAbs has been observed in RA patients compared with the general population (Shiroky et al., 1993; Chan, Al-Saffar & Bucknall, 2001; Raterman et al., 2008; Del et al., 2003; Pan, Gu & Shan, 2015), and AITD and TAbs have been found to be associated with RA disease activity (Koszarny et al., 2013; Joshi et al., 2017). Moreover, it is not rare to find arthritis or synovitis resembling RA in some patients with AITD (Punzi et al., 1997), and the presence of TAbs has been found in synovial fluid even prior to their detection in the serum (Punzi et al., 1991). However, to our knowledge, few data are available on the incidence of TAbs in Chinese RA patients, and studies on the association between TAbs and joint damage as well as synovitis in RA patients remain sparse. Do RA patients with positive TAbs run a higher risk of joint damage or experience more severe synovitis than patients without positive TAbs? Is the clustering of positive TAbs and some RA characteristics associated with an amplified joint damage risk? To minimize the risk of selection bias, we performed a consecutive cohort study recruiting hospitalized RA patients to better evaluate the incidence of TAbs in Chinese RA patients and to investigate whether positive TAbs are linked with joint damage and synovitis in RA patients.

## Materials and Methods

### Patients

Between February 2015 and August 2016, hospitalized patients who fulfilled the 1987 revised criteria of the ACR (Arnett et al., 1988) or the 2010 ACR/European League Against Rheumatism (EULAR) criteria for the classification of RA (Aletaha et al., 2010) were consecutively recruited in the Department of Rheumatology at Sun Yat-Sen Memorial Hospital, Guangzhou, China. All patients were aged ≥ 18 years. The exclusion criteria included the following: individuals overlapping with other autoimmune diseases (e.g., systemic lupus erythematosus, scleroderma, polyarteritis nodosa, dermatomyositis); with hypothalamus or pituitary disease; with serious infection, organ failure, or malignancy; with a history of surgical removal of the thyroid gland; or treated with iodine-containing drugs (e.g., amiodarone, lithium, carbamazepine, phenytoin sodium, interferon-alpha). Written informed consent was obtained from all patients. This study was approved by the Medical Ethics Committee of Sun Yat-Sen Memorial Hospital (identifier: SYSEC-2009-06 and SYSEC-KY-KS-011).

### Clinical assessments

The clinical data of the RA patients were collected at enrollment, as described before (Ma et al., 2015), including the 28-joint tender and swollen joint counts (28TJC and 28SJC, both 0–28), patient and provider global assessments of disease activity (PtGA and PrGA, both 0–10 cm, 10 = worst status), pain visual analog scale (Pain VAS, 0–10 cm, 10 = most pain), Chinese-language version of the Stanford Health Assessment Questionnaire (HAQ, 0–3, 3 = most functional disability; functional limitation was defined as an HAQ score >1), erythrocyte sedimentation rate (ESR, mm/h, normal range: 0–20 mm/h (female), 0–15 mm/h (male)), level of C-reactive protein (CRP, mg/dl, normal range: 0–0.5 mg/dl), level of serum rheumatoid factor (RF, IU/ml, determined by nephelometry (Siemens Healthcare Diagnostics, Munich, Germany), normal range <20 IU/ml), and level of anti-cyclic citrullinated peptide antibody (ACPA, measured by ELISA (Version 2.0, Aesku Diagnostics, Wendelsheim, Germany), normal range <18 U/ml). Disease activity was assessed with DAS28-ESR (the disease activity score in 28 joints based on four variables, including the ESR), DAS28-CRP (the disease activity score in 28 joints based on four variables, including CRP), the simplified disease activity index (SDAI), the clinical disease activity index (CDAI), and the Routine Assessment of Patient Index Data 3 (RAPID3) (Anderson et al., 2012). Disease activity defined by DAS28-ESR was divided into four grades: >5.1 (high disease activity (HDA)), ≥3.2 and <5.1 (moderate disease activity (MDA)), ≥ 2.6 and <3.2 (low disease activity (LDA)), and <2.6 (remission).

### Radiographic assessments

Plain radiographs of the bilateral hands and wrists (anteroposterior view) of all included patients were performed to determine the radiographic status at baseline. Joint damage, including the Sharp/van der Heijde-modified total Sharp score (mTSS), joint erosion (JE), and joint space narrowing (JSN), was scored by two experienced observers (MJD from the Department of Rheumatology and YZH from the Department of Radiology) who were blinded to the patients’ clinical data as described previously (Van der Heijde, 2000). Reliability and agreement were assessed based on the intra-class correlation coefficient (ICC): the mean ICC for interobserver agreement was 0.90. Bony erosion was defined as a cortical break by radiography (Van der Heijde et al., 2013). Patients with an mTSS score >10 were defined as having radiographic joint damage (RJD) (Baker et al., 2011).

### Chemiluminescent immunoassay for detection of serum TAbs and thyroid function

Blood was obtained from all the included RA patients after overnight fasting, and serum specimens were separated and stored at −80 °C for further analysis. The levels of TPOAbs (normal range: 0–60 IU/ml), TgAbs (normal range: 0–60 IU/ml), free thyroxine (FT4, normal range: 11.5–22.7 pmol/L), free triiodothyronine (FT3, normal range: 3.5–6.5 pmol/L), and thyroid-stimulating hormone (TSH, normal range: 0.55–4.78 pmol/L) were measured using chemiluminescent immunoassay (CLIA, Siemens Healthineers, Massachusetts, USA) according to the manufacturer’s instructions. RA patients with positive TPOAbs or TgAbs were assigned to the TAbs-positive group, and patients with neither positive TPOAbs nor positive TgAbs were assigned to the TAbs-negative group.

### Synovial tissues and immunohistochemistry

Synovium was collected by closed Parker-Pearson needle biopsy from one actively inflamed knee joint in RA patient (Schumacher & Kulka, 1972). As described previously (Ma et al., 2014; Zou et al., 2013), serial sealed sections of qualified synovial samples were stained with hematoxylin and eosin (H&E) and also immunohistochemically stained with the commercial mouse monoclonal antibodies (Invitrogen, Carlsbad, CA, USA) according to standard staining protocols: anti-CD3 (clone PS1, T cells), anti-CD15 (clone My1, neutrophils), anti-CD20 (clone L26, B cells), anti-CD34 (clone QB End/10, vascular endothelial cells), anti-CD38 (clone SPC32, plasma cells), and anti-CD68 (clone KP1, macrophages). Irrelevant isotype was used as a negative control. Appropriate positive controls were included to rule out the possibility of an absence of staining due to technical failure.

### Synovitis assessments

At least three tissue pieces that contained well-defined synovial lining and sublining areas were included in the analysis for each specimen. Histological changes in H&E-stained sections were graded according to Krenn’s synovitis score (Krenn et al., 2006). The three-component synovitis score included enlargement of the synovial lining layer, increased density of sublining resident cells, and inflammatory infiltration. Each feature was scored from 0–3, and the total synovitis score ranged from 0–9. RA patients were then divided into high-grade (>4) and low-grade (≤4) synovitis groups according to the synovitis score. Synovitis assessments were performed using a Leica DM2500 microscope (Leica Corp., Heidelberg, Germany) by two independent trained investigators (MYQ and LJZ, both from the Department of Rheumatology) who were blinded to the clinical details of the specimens. Differences between the two investigators were resolved by mutual agreement. The densities of cells with positive staining for CD3, CD15, CD20, CD38, and CD68 and the microvessel counts (MVCs, as confirmed based on the presence of CD34-positive endothelial cells and vessel lumen diameter ≤8 red blood cell diameter) were determined by manual counting. Because each high-power field revealed a synovial area of 0.11740 mm^2^, the densities of cells with positive staining for CD3, CD15, CD20, CD38, CD68, and MVCs were assessed in nine different fields at a magnification of × 400 for a total area of nearly 1 mm^2^; the densities are given as cells per mm^2^.

### Statistical analysis

IBM SPSS Statistics version 20.0 software (IBM, Armonk, NY, USA) was used for the statistical analyses. For continuous variables, descriptive statistics (median, interquartile range (IQR)) were calculated, and the Mann–Whitney analysis of variance on ranks between two groups was used. For categorical variables, indicators are presented as frequencies and percentages, and the Chi-square test or Fisher’s exact test was used. Univariate and multivariate logistic regression analyses were performed, and odds ratios (ORs) and 95% confidence intervals (CIs) were calculated to identify risk factors for RJD. Variables were included in the equation when *p* < 0.05 or removed when *p* > 0.10 following the step-forward selection rule. Spearman’s rank order correlation test was used to assess the relationship between TAb levels and RA duration. A two-tailed *p* < 0.05 was considered statistically significant.

## Results

### Demographic characteristics of RA patients

There were 141 RA patients who fulfilled the inclusion criteria of this study. One patient who was being treated with amiodarone, five patients who were simultaneously diagnosed with malignancies (one hepatocellular carcinoma, one esophageal carcinoma, one breast carcinoma, one nasopharyngeal carcinoma, and one osteosarcoma), and 10 patients who overlapping with other autoimmune diseases (four systemic lupus erythematosus, three scleroderma, two polyarteritis nodosa, one dermatomyositis) were excluded. Finally, a total of 125 RA patients were included in the statistical analysis, of whom 96 (77%) were women. The median disease duration was 60 months, ranging from 12–120 months. Among the included patients, 72% were RF positive, 71% were ACPA positive, and 93% of the patients had bony erosion at baseline. In total, 59 (47%) patients were glucocorticosteroids (GCs) or disease-modifying antirheumatic drugs (DMARDs) therapy naive since six months before enrollment (Table 1).

**Table 1:** Comparison of demographic and clinical features between RA patients with and without positive TAbs.

Parameter	Total (*n* = 125)	TAbs-positive group (*n* = 44)	TAbs-negative group (*n* = 81)	*p*^a^
**Demographic characteristics**				
Women, *n* (%)	96(77)	37(84)	59(73)	0.155
Age (years)	52(46–61)	51(42–58)	54(48–62)	0.071
Disease duration (months)	60(12–120)	72(24–120)	50(12–120)	0.235
Short (<6 months), *n* (%)	13(10)	3(7)	10(12)	0.334
Intermediate (6–24 months), *n* (%)	20(16)	7(16)	13(16)	0.984
Long (>24 months), *n* (%)	92(74)	34(77)	58(72)	0.492
Age of onset (years)	46(39–53)	42(35–49)	47(41–55)	0.025
Smoking, *n* (%)	19(15)	7(16)	12(15)	0.871
**Disease characteristics**				
TJC28	6(2–12)	9(3–13)	5(1–11)	0.040
SJC28	4(1–10)	6(2–11)	3(1–8)	0.074
Pain VAS	4(2–6)	4(3–6)	4(2–6)	0.217
PtGA	5(3–7)	5(3–7)	4(3–7)	0.288
PrGA	5(3–6)	6(3–7)	4(2–6)	0.081
HAQ	0.75(0.19–1.25)	0.88(0.28–1.25)	0.63(0.13–1.06)	0.096
Functional limitation, *n* (%)	38(30)	18(41)	20(25)	0.060
CRP (mg/L)	26.5(7.8–50.9)	24.3(9.8–42.3)	30.0(5.1–56.4)	0.576
ESR (mm/h)	60(37–88)	66(42–90)	58(35–85)	0.399
RF positivity, *n* (%)	90(72)	38(86)	52(64)	0.008
RF titer ≥ 3 ULN, *n* (%)	76(61)	34(77)	42(52)	0.005
ACPA positivity, *n* (%)	89(71)	32(73)	57(70)	0.781
ACPA titer ≥ 3 ULN, *n* (%)	71(57)	26(59)	45(56)	0.703
DAS28-CRP	4.83(3.55–5.68)	5.10(3.89–5.75)	4.37(3.49–5.60)	0.076
DAS28-ESR	5.45(4.26–6.48)	6.00(4.97–6.57)	4.93(4.18–6.47)	0.036
SDAI	21(10–33)	25(16–37.8)	18(9–31)	0.084
CDAI	24.4(13.8–36.8)	27.1(16.3–37.8)	21.3(13.1–34.4)	0.049
RAPID3	4.03(1.97–5.39)	4.39(2.93–5.63)	3.75(1.78–5.09)	0.155
**Radiographic status**				
Bony erosion, *n* (%)	116(93)	40(91)	76(94)	0.547
JNS subscore	3(0–16)	8(0–26)	2(0–11)	0.088
JE subscore	9(3–23)	13(3–34)	8(3–19)	0.075
mTSS	11(4–37)	19(5–62)	9(4–31)	0.076
RJD, *n* (%)	64(51)	30(68)	34(42)	0.005
**Previous medications, *n* (%)**				
Naive^b^	59(47)	18(41)	41(51)	0.299
GCs	52(42)	20(46)	32(40)	0.519
MTX	35(28)	13(30)	22(27)	0.777
LEF	24(19)	7(16)	17(21)	0.491
SASP	7(6)	2(5)	5(6)	0.705
HCQ	10(8)	4(9)	6(7)	0.740
CysA	2(2)	0(0)	2(3)	NA
Biologics	5(4)	2(5)	3(4)	0.819

**Notes.**

a Comparison between the TAbs-positive group and the TAbs-negative group. Data are described as the median (interquartile range) unless stated otherwise.

b Without glucocorticosteroids or disease-modifying antirheumatic drugs therapy within the previous six months.

GCs glucorticosteroids MTX methotrexate LEF leflunomide SASP sulfasalazine HCQ hydroxychloroquine CysA cyclosporin A NA not applicable

**Table 2:** Thyroid abnormalities in RA patients with and without positive TAbs.

Parameter	Total (*n* = 125)	TAbs-positive group (*n* = 44)	TAbs-negative group (*n* = 81)	*p*^a^
**Thyroid function**				
FT3 (pmol/L)	4.23(3.70–4.91)	4.22(3.67–4.61)	4.31(3.71–4.98)	0.254
FT3 abnormality, *n* (%)	14(12)	9(18)	5(6)	0.019
FT3 elevated	2(2)	2(2)	0(0)	NA
FT3 reduced	12(10)	7(16)	5(6)	0.076
FT4 (pmol/L)	15.96(14.70–18.21)	15.25(14.35–18.02)	16.18(14.92–18.39)	0.199
FT4 abnormality, *n* (%)	8(14)	6(14)	2(3)	0.022
FT4 elevated	4(7)	3(7)	1(1)	0.125
FT4 reduced	4(7)	3(7)	1(1)	0.125
TSH (pmol/L)	1.09(0.55–2.12)	1.22(0.54–2.36)	1.01(0.55–1.99)	0.380
TSH abnormality, *n* (%)	10(8)	8(18)	2(3)	0.004
TSH elevated	5(4)	4(9)	1(1)	0.052
TSH reduced	5(4)	4(9)	1(1)	0.052
**Thyroid disorders, *n* (%)**				
Hyperthyroidism	5(4)	4(9)	1(1)	0.052
Clinical hyperthyroidism	3(2)	3(7)	0(0)	NA
Subclinical hyperthyroidism	2(2)	1(2)	1(1)	0.582
Hypothyroidism	5(4)	4(9)	1(1)	0.052
Clinical hypothyroidism	2(2)	2(5)	0(0)	NA
Subclinical hypothyroidism	3(2)	2(5)	1(1)	0.283

**Notes.**

a Comparison between the TAbs-positive group and the TAbs-negative group. Data are described as the median (interquartile range) unless stated otherwise.

### Thyroid abnormalities in RA patients

The profile of thyroid abnormalities is demonstrated in Table 2. Among the patients, 35 (28%) were positive for TPOAbs, and 27 (22%) were positive for TgAbs, with 44 (35%) patients having either positive TPOAbs or positive TgAbs and 17 (14%) being positive for both. TPOAbs positivity in women was 31%, and TgAbs positivity was 26%. Additionally, significantly higher prevalences of TPOAbs and TgAbs were found in patients with seropositive RF versus those with seronegative RF (36% vs. 9% and 27% vs. 9%, respectively; both *p* < 0.05). Five (4%) patients were diagnosed with hypothyroidism (two (2%) had clinical hypothyroidism, and three (2%) had subclinical hypothyroidism), and five (4%) patients had hyperthyroidism (three (2%) showed clinical manifestations, and two (2%) showed subclinical hyperthyroidism). All patients with thyroid disorders were in the TAbs-positive group except for 2 patients (1 subclinical hyperthyroidism and 1 subclinical hypothyroidism) in the negative group. Accordingly, FT3, FT4, and TSH abnormalities were all significantly more frequent in the TAbs-positive group than in the TAbs-negative group (21% vs. 6%, 14% vs. 3%, and 18% vs. 3%, respectively; all *p* < 0.05; Table 2).

### Comparison of RA characteristics between patients with and without positive TAbs

There were 44 (35%) patients in the TAbs-positive group. A significantly greater percentage of patients with RJD was observed in the TAbs-positive group versus the TAbs-negative group (68% vs. 42%, *p* = 0.005; Table 1). Compared with the TAbs-negative group, patients with positive TAbs had RA onset at a significantly younger age (42(35–49) years vs. 47(41–55) years, *p* = 0.025). RF positivity and disease activity indicators, including TJC28, DAS28-ESR, and CDAI, were significantly higher in the TAbs-positive group (all *p* < 0.05), with borderline significant differences in SJC28, PrGA, DAS28-CRP, and SDAI as well as in the percentage of functional limitation. Similarly, a significantly higher percentage of patients with HDA was seen in the TAbs-positive group versus the TAbs-negative group (68% vs. 49%, *p* = 0.043). However, there was no significant difference in other RA clinical features between the two groups, including gender, disease duration, smoking status, and previous medications used since 6 months before enrollment (all *p* > 0.05; Table 1).

### Comparison of synovitis between patients with and without positive TAbs

**Figure 1:**
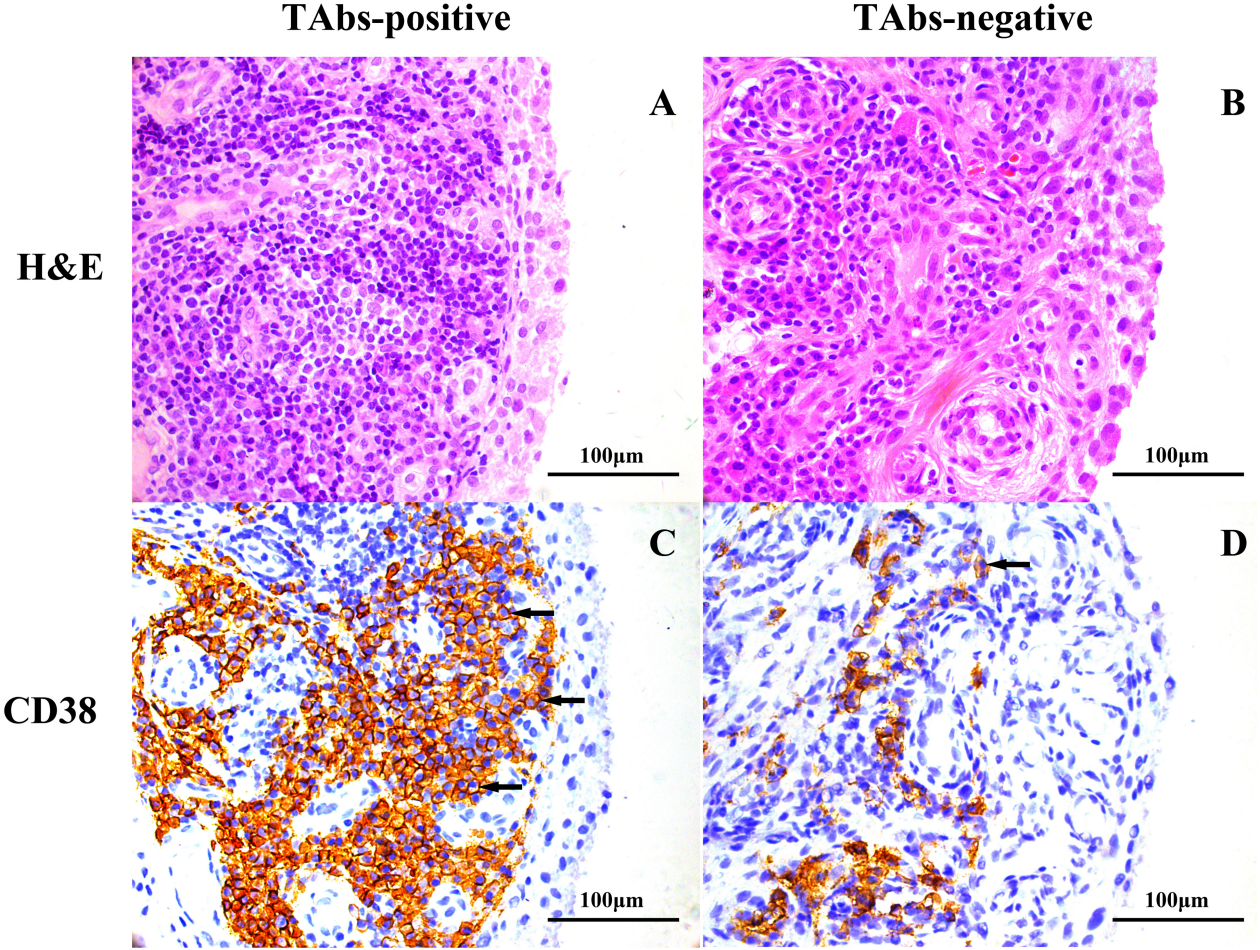
Representative images of H&E and immunohistochemical staining for CD38 in the synovium of RA patients based on TAb status. H&E staining, high-grade synovitis (Krenn’s synovitis score = 6) in a TAbs-positive RA patient (A) and low-grade synovitis (Krenn’s synovitis score = 3.5) in a TAbs-negative RA patient (B); immunohistochemical staining, expression of CD38 in synovium of a TAbs-positive RA patient (C) and a TAbs-negative RA patient (D). Significantly more pronounced amounts of CD 38-positive plasma cells infiltrated the TAbs-positive synovium versus the TAbs-negative control. The black arrows point to CD38-positive cells located at the sublining area of the synovium. (A–D), original magnification, ×400.

A total of 22 patients had qualified synovial tissues, of whom eight were patients with positive TAbs. Notably, RF positivity and ACPA positivity were both 100% in the eight patients, and were 71% and 79% respectively in the 14 patients with negative TAbs. Significantly more pronounced infiltration of CD38-positive plasma cells was observed in the TAbs-positive synovium (1,354(847–2,096) cells/mm^2^) than in the TAbs-negative control (274(109–1,252) cells/mm^2^) (*p* = 0.048; Table 3). The percentage of patients with high-grade synovitis was also higher in the TAbs-positive group than in the TAbs-negative group (63% vs. 36%), but the difference was not significant (*p* = 0.221). Representative images of H&E and immunohistochemical staining for CD38 in the synovium of RA patients with and without positive TAbs are shown in Fig. 1. No significant difference between the two groups was observed in the densities of other infiltrated inflammatory cells, including CD3-positive T cells, CD15-positive neutrophils, CD20-positive B cells, CD68-positive macrophages, or MVCs (all *p* > 0.05; Table 3).

**Table 3:** Synovial histological features in RA patients with and without positive TAbs.

Parameter	TAbs-positive group (*n* = 8)	TAbs-negative group (*n* = 14)	*p*^a^
MVC, /mm^2^	185(87–223)	135(89–168)	0.339
CD3+ T cells, /mm^2^	763(242–1,404)	615(359–1,268)	1.000
CD15+ neutrophils, /mm^2^	410(103–614)	340(120–637)	0.785
CD20+ B cells, /mm^2^	785(144–1,058)	385(208–2,054)	0.785
CD38+ plasma cells, /mm^2^	1,354(847–2,096)	274(109–1,252)	0.048
CD68+ macrophages, /mm^2^	1,540(1,020–1,818)	1,003(511–1,681)	0.195
Krenn’s synovitis score	5.0(2.5–6.8)	4.0(2.8–5.0)	0.267
Low-grade synovitis, *n*(%)	3(37)	9(64)	0.221
High-grade synovitis, *n*(%)	5(63)	5(36)	–
Hyperplasia of lining layer	2.0(1.3–2.0)	1.0(1.0–2.0)	0.105
Inflammatory infiltration	1.0(1.0–2.0)	1.0(0.8–1.6)	0.486
Synovial stroma activation	2.0(1.0–2.0)	1.3(1.0–2.0)	0.439

**Notes.**

a Comparison between the TAbs-positive group and the TAbs-negative group. Data are described as the median (interquartile range) unless stated otherwise.

### Risk factors for RJD in RA patients

**Figure 2:**
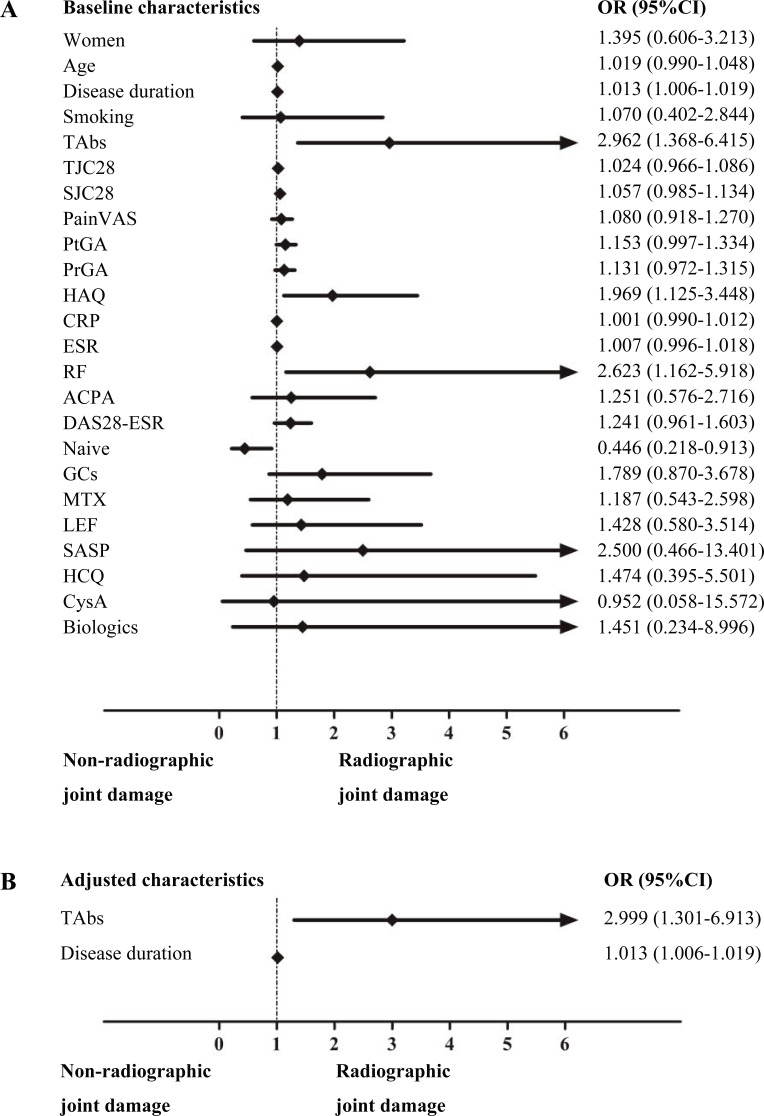
Logistic regression analysis of the risk factors for RJD in RA patients. Univariate logistic regression analysis of the risk factors for RJD (A); multivariate logistic regression of the risk factors for RJD after adjusting for confounding factors as mentioned above in the univariate logistic regression analysis (B).

Logistic regression analysis was performed to investigate the risk factors for RJD (Fig. 2). Univariate logistic regression analysis performed on demographic and disease characteristics as well as TAbs status showed that medication-naive status was a protective factor (OR 0.446, 95% CI [0.218–0.913]; *p* = 0.027), whereas a longer disease duration (OR 1.013, 95% CI [1.006–1.019]; *p* < 0.001), a higher HAQ score (OR 1.969, 95% CI [1.125–3.448]; *p* = 0.018), positive RF (OR 2.623, 95% CI [1.162–5.918]; *p* = 0.020), and positive TAbs (OR 2.962, 95% CI [1.368–6.415]; *p* = 0.006) were risk factors for RJD. Multivariate logistic regression analysis was performed to control for confounding factors. The result revealed that positive TAbs (OR 2.999, 95% CI [1.301–6.913]; *p* = 0.010) and disease duration (OR 1.013, 95% CI [1.006–1.019]; *p* < 0.001) were independently associated with RJD. No significant correlations were found neither between TPOAb levels and disease duration (*r* = 0.082, *p* = 0.366) nor TgAb levels and disease duration (*r* =  − 0.057, *p* = 0.530).

Further analysis of positive TAbs yielded an OR of 2.845 (95% CI [1.062–7.622]) for RJD when comparing women in the TAbs-positive group (*n* = 37) with those in the TAbs-negative group (*n* = 59) (*p* = 0.038). Subgroup studies revealed that a high titer of ACPA (defined as an ACPA level ≥ 3 times the upper limit of the normal range (ULN)) was also a risk factor for RJD (OR 2.107, 95% CI [1.025–4.328]; *p* = 0.043). After adjusting for confounding factors, as mentioned above for the univariate logistic regression analysis, a high titer of ACPA remained significantly associated with RJD (OR 2.467, 95% CI [1.076–5.655]; *p* = 0.033).

## Discussion

This study demonstrated that the prevalence of TAbs is 35% and positive TAbs are independently associated with RJD in RA patients. There was significantly more pronounced infiltration of CD38-positive plasma cells in the TAbs-positive synovium than in the negative control. Additionally, RA patients with positive TAbs had RA onset at a younger age, and higher TJC28 as well as disease activity was also observed in patients with positive TAbs than in those without positive TAbs. The results importantly indicated a significant association between positive TAbs and joint destruction as well as clinical disease activity in RA, possibly due to more infiltration of plasma cells in the TAbs-positive synovium. To the best of our knowledge, this is the first study to obtain evidence that joint destruction might be amplified in RA patients with an elevated presence of TAbs.

It is well recognized that patients suffering from an autoimmune disease have a strong hereditary susceptibility to other autoimmune diseases (Roldan, Alonso & Barrio, 1999). In the previous study, a total of 15,007 adults subjected to health checkups with TAbs and thyroid function testing were recruited from Peking Union Medical College Hospital (Wen et al., 2015). Compared with this Chinese healthy control cohort, significantly higher incidences of both TPOAbs and TgAbs were observed in RA patients (28% vs. 9% and 22% vs. 10%, respectively; both *p* < 0.001), with a similar tendency found in women (31% vs. 14% and 26% vs. 16%, respectively; both *p* < 0.001). A meta-analysis including 1021 RA patients and 1,500 healthy controls showed that TAbs positivity in patients with RA was higher than in healthy controls (TgAbs: OR 3.17, 95% CI [2.24–4.49]; TPOAbs: OR 2.33, 95% CI [1.24–4.39]) (Pan, Gu & Shan, 2015). A large cohort study including 800 RA patients from Colombia also showed that the incidence of TAbs was 37.8% for TPOAbs and 20.8% for TgAbs. Further literature review disclosed geographical variation in TAbs positivity and showed that the prevalence ranges from 5–37% for TPOAbs, from 6-31% for TgAbs, and from 11.4–32% for either (Cardenas et al., 2012). However, several other studies showed no difference in the prevalence of TAbs in RA patients compared with the general population (McCoy et al., 2012; Przygodzka & Filipowicz-Sosnowska, 2009). Given the striking discrepancy between these results, controversy persists over this issue. Although different methods for testing for TAbs in different studies and differences in iodine intake between different areas might explain some of the discrepancy, it is reasonable to propose that the presence of TAbs may play a specific role in RA development and/or progression. In the current study, higher incidences of TPOAbs and TgAbs were found in RA patients compared with the reportedly healthy controls, and a similar result was found in women, which was in accordance with most previous studies. Although no correlation between RF titer and TAbs positivity was observed (Yavasoglu et al., 2009), our results indicated a significantly higher prevalence of both TPOAbs and TgAbs in RA patients with seropositive RF than in those without seropositive RF, which further strengthens the hypothesis of an association between positive TAbs and RA. However, this result deserves further consideration because several cases have been reported showing false-positive results in Tg assays due to interference by RF resulting from its heterophile ability to bind to other Fc-region IgG antibodies (Massart, Corcuff & Bordenave, 2008; Astarita et al., 2015).

The association between thyroid abnormalities and RA disease activity has been extensively studied. Thyroid dysfunction has been reported to be linked with a longer duration and an increased prevalence of morning stiffness in RA patients, and disease activity can be greatly improved by correction of the hypothyroid status (Delamere, Scott & Felix-Davies, 1982). Joshi et al. (2017) showed that RA patients with hypothyroidism had higher DAS28-ESR, TJC28, and Pain VAS scores, and significant correlations were observed between TSH levels and ESR as well as DAS28-ESR. Recently, Koszarny et al. (2013) revealed significant positive correlations between TPOAbs and DAS28 and between TgAbs and inflammatory markers, including ESR and CRP; moreover, a higher mean DAS28 was observed in TPOAbs-positive versus TPOAbs-negative patients as well as in TgAbs-positive versus TgAbs-negative patients. In addition, data from a population-based case-control study of incident RA cases (1,998 adult cases, 2,252 controls) revealed a significant association between thyroxin substitution (reflecting an autoimmune thyroid disease) and the risk of developing either ACPA-positive or ACPA-negative RA (Bengtsson et al., 2014). Nevertheless, debate still exists because several studies showed no correlation between thyroid abnormalities and RA disease activity (Atzeni et al., 2009; Cardenas et al., 2012). In the present study, TJC28 and disease activity scores, including DAS28-ESR and CDAI, were significantly higher in patients with positive TAbs than in patients without positive TAbs. Moreover, RA patients with positive TAbs had an increased risk of earlier RA onset. This phenomenon might occur because some TAbs are induced by chronic inflammation in RA patients. Conversely, it could also be reasonable to speculate that positive TAbs may play a role as a trigger or an accelerator in RA development. The observation of Blake et al. (1979) suggests that thyroid autoantibodies may be produced locally in the joints, and some patients have been reported to develop thyroiditis during follow-up (Punzi et al., 1991). Thus, it is possible that a higher TAbs titer may be produced due to more plasma cells or lymphocytes infiltrating the synovial membrane of RA patients with positive TAbs. Accordingly, in the present study, significantly more pronounced numbers of plasma cells infiltrated the synovial tissues of patients in the TAbs-positive group, and a higher percentage of patients with high-grade synovitis was also observed in the TAbs-positive group, although this difference was not significant. Therefore, the presence of more plasma cells in the TAbs-positive synovial tissue might contribute to joint inflammation or destruction. The small size of the qualified synovial samples might explain the lack of significant differences in other indicators of synovitis. Most importantly, although few studies have reported a correlation between thyroid abnormalities and RA joint destruction (Cardenas et al., 2012), our results revealed that positive TAbs were independently associated with joint destruction in RA patients, which possibly suggested a detrimental role for TAbs in joint damage in RA. The mechanisms by which thyroid abnormalities may be linked with RA have not been fully determined yet; however, explanations are suggested by the shared genetics that determine or influence autoantigen presentation and the regulation of the immune response. Indeed, the shared HLA-DRB1 epitope has been demonstrated to be a risk factor not only for RA development but also for joint damage in RA patients, independent of ACPA status (Rojas-Villarraga et al., 2009; Suzuki et al., 2013). Hence, the results obtained in the current study may be partly attributed to sharing of the HLA-DRB1 epitope in RA patients with concurrent positive TAbs. In conclusion, the associations between an elevated presence of TAbs and amplified joint damage, more pronounced infiltration of plasma cells in the synovium, as well as higher levels of disease activity indicators suggest a probably significant and robust effect exerted by positive TAbs, independent of the definition of RJD.

There are several limitations to this study that merit careful consideration. First, data from cross-sectional studies are not sufficiently convincing to draw a clear causal link between the presence of TAbs and joint damage or disease activity in RA. Second, the relatively small number of RA patients and synovial tissues included in this study may have precluded obtaining a meaningful significant difference or a robust conclusion, and further stratification was difficult to achieve. Third, a general population without RA should have been recruited simultaneously as a control group to improve the data analysis. Further studies are needed in future to explore the mechanism of abnormal TAb levels in RA and to confirm their association with joint destruction in a large and prospective case-control study.

## Conclusions

Our data showed that positive TAbs were significantly associated with RJD in RA patients. Joint destruction may be amplified in RA patients with an elevated presence of TAbs, which supports the importance and necessity of TAbs and thyroid function screening and monitoring in RA patient management in clinical practice. RA patients with positive TAbs should thus be paid more attention regarding joint destruction or even joint progression during follow-up.

##  Supplemental Information

10.7717/peerj.4216/supp-1 Supplemental Information 1 Data of 125 included RA patients A total of 125 RA patients were included in the statistical analysis.

10.7717/peerj.4216/supp-2 Supplemental Information 2 Data of 22 RA patients with synovial tissues A total of 22 patients had qualified synovial tissues, of whom 8 were patients with positive TAbs.

## Additional Information and Declarations

### Data Availability

The following information was supplied regarding data availability: The raw data have been provided in the Supplemental Files.
